# Estrogen weakens muscle endurance via estrogen receptor-p38 MAPK-mediated orosomucoid (ORM) suppression

**DOI:** 10.1038/emm.2017.307

**Published:** 2018-03-30

**Authors:** Yang Sun, Zhen Qin, Jing-Jing Wan, Peng-Yuan Wang, Yi-Li Yang, Jian-Guang Yu, Bo-Han Hu, Ding-Feng Su, Zhu-Min Luo, Xia Liu

**Affiliations:** 1 Department of Pharmacology, School of Pharmacy, Second Military Medical University, Shanghai, People’s Republic of China; 2 Suzhou Institute of Systems Medicine, Center for Systems Medicine, Chinese Academy of Medical Sciences, Suzhou, People’s Republic of China

## Abstract

Gender differences in fatigue manifest as females being more prone to feel exhaustion and having lower muscle endurance. However, the mechanisms of these effects remain unclear. We investigated whether orosomucoid, an endogenous anti-fatigue protein that enhances muscle endurance, is involved in this regulation. Female rats exhibited lower muscle endurance, and this gender difference disappeared in orosomucoid-1-deficient mice. Female rats also exhibited weaker orosomucoid induction in serum, liver and muscle in response to fatigue compared with male rats. Ovariectomy elevated orosomucoid levels and increased swimming time, and estrogen replenishment reversed these effects. Exogenous estrogen treatment in male and female mice produced opposite effects. Estrogen decreased orosomucoid expression and its promoter activity in C2C12 muscle and Chang liver cells *in vitro*, and estrogen receptor or p38 mitogen-activated protein kinase blockade abolished this effect. Therefore, estrogen negatively regulates orosomucoid expression that is responsible for the weaker muscle endurance in females.

## Introduction

Fatigue generally manifests as a reversible decline in performance by affected individuals,^1^ and it is divided into central fatigue and physical fatigue. Patients with central fatigue, especially chronic fatigue syndrome, always complain about reduced physical endurance,^2, 3^ and physical fatigue is generally accompanied by deceased muscle function and fuel depletion. Muscle fatigue is a common phenomenon that is defined as an exercise-induced reduction in the ability of a muscle or muscle group to generate maximal force^4^ that limits daily life, athletic performance or other strenuous or prolonged activity. Muscle endurance is usually used to evaluate muscle resistance to fatigue that refers to the capacity of a muscle or a group of muscles to sustain repeated contractions against a resistance (for example, a given load or repetitive stimuli) for an extended period of time. Fatigue index is used to measure muscle endurance.^5, 6^

Gender difference in fatigue and muscle endurance is an important factor in clinical practice.^7, 8^ Females are more prone to complain of fatigue and require greater recovery time than males performing the same intensity of work.^9^ Most studies have reported that 75% or more of patients with chronic fatigue syndrome were female.^7, 10^ Females suffering diseases, such as rheumatoid arthritis,^11^ poststroke^8^ and acute myocardial infarction,^12^ are significantly more likely to present with fatigue, and their fatigue degree is more serious than that of males. Lower endurance test scores were also found for women than for men in healthy people and in patients, especially in athletes.^5, 6, 13, 14, 15, 16^ These studies are consistent with the fact that female sports records are not as good as those of males, and female work endurance is generally shorter than that of males. These gender differences likely protect females from being overworked and suggest that some hormonal factors, particularly estrogen, are involved in this phenomenon. However, no direct evidence or an underlying mechanism has been clearly established.

Our previous studies reported that the endogenous anti-fatigue protein orosomucoid (ORM) enhanced muscle endurance. ORM, also known as α-1 acid glycoprotein, is an acute-phase protein that exhibits a molecular weight of 37−54 kDa, a low pI of 2.8−3.8 and heavy glycosylation (45%). The liver primarily synthesizes ORM, but many extrahepatic tissues also produce ORM under various physiological and pathological conditions. ORM exhibits many biological activities, including modulating immunity, binding and carrying drugs, maintaining the capillary barrier and acting as a disease marker.^17^ The role of ORM in cancer,^18, 19, 20^ cardiovascular diseases^21, 22^ and energy metabolism^23^ was revealed recently. We previously reported that ORM was significantly elevated in the sera, liver and muscle tissues of fatigued rodents (for example, sleep-deprivation, forced swimming and treadmill-running models) and the sera of chronic fatigue syndrome patients.^2, 24^ Further studies demonstrated that ORM functioned as an anti-fatigue protein via activation of muscle CCR5 (C-C chemokine receptor type 5) to increase glycogen storage and muscle endurance.^2^ Therefore, we examined whether ORM participated in the differential regulation of fatigue and muscle endurance in males and females.

We found that weaker muscle endurance in females was closely related to reduced ORM induction in response to fatigue. The *in vivo* and *in vitro* data demonstrated that estrogen could downregulate ORM expression via estrogen receptors (ERs) and the p38 mitogen-activated protein kinase (MAPK) pathway that decreased muscle endurance. This difference in ORM may be responsible for the gender difference in muscle fatigability.

## Materials and methods

### Reagents

17β-Estradiol and ICI 182780 (a selective ER degrader) were purchased from Sigma-Aldrich (St Louis, MO, USA). BEZ235 (phosphatidylinositol 3-kinase (PI3K) inhibitor) and SB203580 (MAPK inhibitor) were purchased from Selleck Chemicals (Houston, TX, USA). The antibody against ORM (rat) was purchased from Abcam (Cambridge, UK), and the antibody against ORM (mouse) was obtained from Genway (San Diego, CA, USA). An antibody against glyceraldehyde-3-phosphate dehydrogenase (GAPDH) was purchased from the Beyotime Institute of Biotechnology (Shanghai, China). Secondary antibodies conjugated with IRDye 800CW were purchased from Rockland Immunochemicals (Limerick, PA, USA).

### Animals

Sprague-Dawley rats weighing 220±7 g and C57BL/6 mice weighing 22±2 g were purchased from Sino-British SIPPR/BK Laboratory Animals (Shanghai, China). All animals were re-weighed and weight-matched before experiments. ORM1-deficient mice were generated and identified as reported previously.^2, 23^ Generally, ORM1 knockout mice were generated through the construction of vectors targeting exons 1 to 5 of the ORM1 gene, transfection and selection of embryonic stem cells (SCR012) and injection of embryonic stem cells into blastocysts of C57BL/6J mice. The resulting chimerical males were bred with female C57BL/6J mice to produce heterozygous mutant mice that were inter-crossed to obtain homozygous and wild-type mice.

All animals were housed under controlled temperature (22±3 °C) and lighting (0800−2000 h) conditions with free access to water and a standard rodent diet. Experiments were performed in accordance with the National Institute of Health’s ‘Guide for the Care and Use of Laboratory Animals’ and the approval of the Scientific Investigation Board of the Second Military Medical University.

### Cell line culture

C2C12 cells (mouse muscle myoblasts) were purchased from the Shanghai Institutes for Biological Sciences, Chinese Academy of Sciences (Shanghai, China) and cultured in serum-supplemented media composed of Dulbecco’s modified Eagle’s medium and 10% fetal bovine serum. Human Chang liver cells were purchased from BioHermes Biomedical Technology (Wuxi, China) and cultured in RPMI-1640 medium with 10% fetal bovine serum.

### Weight-loaded forced swimming

Experiments were performed as previously described.^2^ Male and female rats were matched for weight that was controlled at 220±7 g. Briefly, mice or rats were placed individually in a water tank (46 cm tall, 20 cm in diameter, 21–23 °C). Experimental animals were forced to swim with a load of 8% of each body weight attached to the proximal end of the tail. The swimming time was measured from the time when swimming began to the time when animals could not return to the water surface 10 s after sinking.^25^

### Sleep-deprivation fatigue

Male and female rats were matched for weight that was controlled at 220±7 g. Rats were deprived of sleep for 5 consecutive days in a cage filled with water to a height of 1.5 cm, as described previously,^2, 26^ and the fatigue degree was evaluated using weight-loaded forced swimming.

### Treadmill-running

Male and female rats were matched for weight that was controlled at 220±7 g. These rats were subjected to running on a treadmill system to exhaustion as described previously.^2^ The rats were forced to run 1 h per day at a speed of 30 m min^−1^ for 1-week adaptation training. The initial speed was 12 m min^−1^, but it gradually increased every 5 min at a rate of 3 m min^−1^ until reaching 30 m min^−1^. Rats were forced to run at a speed of 30 m min^−1^ 1 week later until the animals were unable to maintain the pace of the treadmill for up to 10 s. The rats were considered exhausted when they could not right themselves when placed in a supine position to check. The exhaustion time was recorded.

### Ovariectomy

Female rats were bilaterally ovariectomized as previously described.^27^ In general, animals were intraperitoneally injected with 30 mg kg^−1^ of nembutal for anesthesia. The surgical area from the hip to the lowest rib on both sides was shaved and sterilized. A 1.5 cm incision through the skin, connective tissue and muscle layer was made, and the ovaries were exteriorized with the associated fat pad and fallopian tube. Sham-operated animals (non-ovariectomized) were anesthetized and subjected only to surgery without removal of the ovaries.

### Serum biochemical index

Serum levels of ORM and estradiol were measured using specific enzyme-linked immunosorbent assay (ELISA) kits. The rat ORM ELISA kit was obtained from Abcam. The mouse ORM ELISA kit was obtained from Shanghai Westang Bio-Tech (Shanghai, China). Rat and mouse estradiol (E2) ELISA kits were obtained from Shanghai Westang Bio-Tech Proteins were detected according to the manufacturer’s instructions.

### Western blotting for ORM

Western blotting was performed as previously reported.^28^ Tissues or cells were homogenized on ice and lysed in a lysis buffer that contained a protease inhibitor mixture (Kangchen, Shanghai, China). Protein concentrations were measured using a BCA Protein Assay Kit (Beyotime Institute of Biotechnology). Proteins were separated on sodium dodecyl sulfate–polyacrylamide gels and transferred to a nitrocellulose membrane that was incubated with antibodies specific to ORM. Equal sample loading was confirmed by re-probing the blots for GAPDH.

### Fatigue induction in isolated muscle

Muscle fatigability was measured as previously described.^2^ Mouse and rat soleus muscles were isolated in the integrity without nerve damage. One end of the muscle was fixed, and the other end was linked to a biotic signal collection sensor and processing system (MedLab-U/4CS, MeiYi, Nanjing, China). Evoked contractions of the muscle strips were continuously detected for 3 min under electrical stimulation trains at 10 V lasting 5 ms delivered every second for 3 min. The fatigue index was calculated as the ratios of tensions at 1, 2 and 3 min to the initial tension (average of the first 5 contractions).

### Reverse transcription-PCR (RT-PCR)

RT-PCR was performed as previously reported.^29^ Total RNA was extracted from C2C12 cells using Trizol reagent (Invitrogen, Grand Island, NY, USA) and reverse-transcribed using a PrimeScript RT Master Mix Perfect Real-Time Kit (Takara, Otsu, Japan). Real-time quantitative RT-PCR analysis was performed using SYBR RT-PCR kits (Takara). Expression levels were normalized to the control gene GAPDH. The following primer sequences were used:^23^ ORM, 5′-ACACAATAGAGCTTCGGGAGTC-3′ (forward), 5′-ATATCTGGCCTTTTGGCATAGA-3′ (reverse); and GAPDH, 5′-−GTATGACTCCACTCACGGCAAA-3′ (forward), 5′-GGTCTCGCTCCTGGAAGATG-3′ (reverse).

### Transient transfection and dual luciferase reporter assays

The ORM luciferase plasmid contained a −1.4 kb to −150 bp fragment of the mouse ORM promoter in front of a luciferase reporter gene^30^ that was kindly provided by Yun Sok Lee (School of Biological Sciences, Seoul National University, Seoul, Korea). HEK293 cells were transfected with the luciferase reporter plasmid and incubated with the indicated doses of estrogen for 24 h. Luciferase activities were measured using the Dual-Luciferase Reporter Assay System (Promega, Madison, WI, USA) according to the manufacturer’s instructions.^31^

### Statistical analysis

Student’s *t*-test was used to compare two distinct groups. One-way analysis of variance followed by the least significant difference *t-*test was used to compare more than two groups. Interactions were analyzed using two-way analysis of variance followed by the least significant difference *t* test. Friedman *M*-test was used when the data exhibited heterogeneity of variance. Data are presented as the means±s.e.m. *P*<0.05 was considered statistically significant. Experiments were repeated at least three times. Statistical analyses were performed using JMP 4.0.4 software from the SAS Institute (Cary, NC, USA).

## Results

### Females exhibited lower muscle endurance than males *in vivo* and *in vitro*

**Figure 1:**
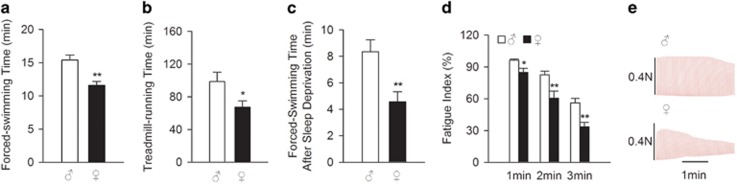
Females exhibit lower muscle endurance than males, as demonstrated by *in vivo* and *in vitro* studies. (**a**) Forced-swimming times of male and female Sprague-Dawley rats (*n*=7–8/group). (**b**) Treadmill-running times of male and female Sprague-Dawley rats after adaptation trainings for 1 week (*n*=6/group). (**c**) Forced-swimming times of male and female Sprague-Dawley rats after 5-day sleep deprivation (*n*=5−6/group). (**d**) Electrically evoked contractions of soleus muscle isolated from male and female Sprague-Dawley rats. The ratio of tension at 1, 2 or 3 min to the initial tension (average of the first 5 contractions) is expressed as the fatigue index. (**e**) Representative records of the fatigue tests in (**d**). Male and female rats were matched for weight. Data are presented as the means±s.e.m. *P<0.05 and **P<0.01 by Student’s *t-*test.

We investigated whether gender difference in fatigue and muscle endurance existed in rodent fatigue models. It is well known that weight-loaded forced-swimming and treadmill-running models are similar to physical fatigue, and the sleep-deprivation model is generally used in the study of chronic fatigue syndrome patients.^32^ Rats were used to evaluate endurance differences in the three fatigue models because of the high mortality rate in rat sleep-deprivation model. Figures 1a and b show that male rats swam or ran much longer than female rats. Male rats also swam much longer than female rats after 5 days of sleep deprivation (Figure 1c). Gender differences in muscle endurance were also measured *in vitro*. Slow twitch type I fibers are highly involved in endurance exercise. Therefore, we used isolated soleus muscles.^33^ Repetitive electrical stimulation induces muscle fatigue that manifests as a decline in muscle contraction ability.^34^ The fatigue index measured muscle endurance. This index was calculated as the ratios of tensions at 1, 2 or 3 min to the initial tension. Female rats exhibited a significantly lower fatigue index in response to continuous electrical stimulation (Figures 1d and e). These results suggest that females are prone to exhaustion and have lower muscle endurance.

### ORM knockout abolished the gender difference in muscle endurance

**Figure 2:**
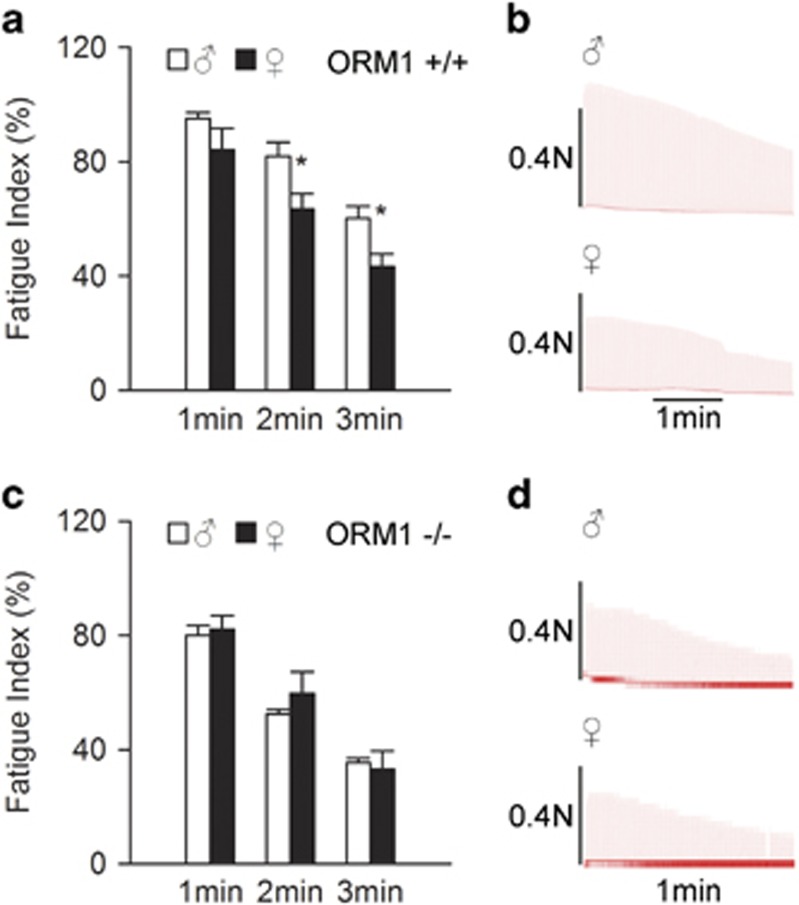
Orosomucoid (ORM) knockout abolished the gender difference in muscle endurance. (**a**, **b**) Representative records of electrically evoked contractions of soleus muscle isolated from ORM1+/+ mice (*n*=6−7 per group). (**c**, **d**) Representative records of electrically evoked contractions of soleus muscle isolated from ORM1−/− mice (*n*=5−6 per group). Data are presented as the means±s.e.m. *P<0.05 by Student’s *t-*test.

ORM is an endogenous anti-fatigue protein.^2^ Therefore, we examined the role of ORM in the gender difference in muscle endurance using ORM-deficient mice. There are two isoforms of ORM in humans (ORM1 and ORM2), one isoform in rats (ORM) and three isoforms in mice (ORM1, ORM2 and ORM3). Human and mouse ORM1s are the predominant serum ORM, and it accounts for most of the changes in the ORM pool size during acute-phase responses.^17^ ORM1 knockout mice were constructed as previously reported.^2^ The muscle fatigue index in ORM1^+/+^ female mice was lower than that in male mice (Figures 2a and b), similar to the results in rats. However, this difference disappeared in ORM1^−/−^ mice (Figures 2c and d), indicating an essential role of ORM in this process.

### Fatigue-induced ORM elevation in females was weaker than in males

**Figure 3:**
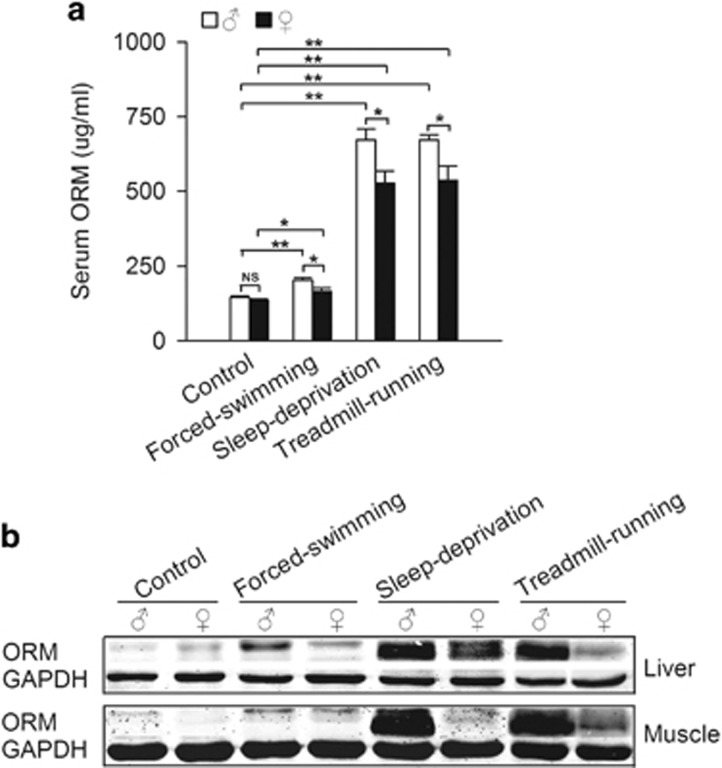
Fatigue-induced orosomucoid (ORM) elevation in females is weaker than males. (**a**) Serum ORM levels of male and female Sprague-Dawley rats in forced-swimming, sleep-deprivation and treadmill-running fatigue models. (**b**) Representative western blots of ORM in liver and muscle tissues derived from the rats mentioned in (**a**) (*n*=6−7/group in control group, *n*=4/group in fatigue models). Data are presented as the means±s.e.m. NS, not significant, *P<0.05 and **P<0.01 by two-way analysis of variance (ANOVA) with least significant difference (LSD) *t-*test.

We examined whether gender differences in fatigue-induced ORM expression existed. Fatigue was induced as described in Figure 1. Fatigue induced a significant increase of ORM in serum, liver and muscle tissues, and this increase was more obvious in the sleep-deprivation and treadmill-running models. Interestingly, the increase was significantly stronger in male rats than in female rats in response to the same fatigue model (Figures 3a and b), suggesting that the gender difference in fatigue-induced ORM induction participates in the gender difference in muscle endurance. Notably, the induced ORM levels in serum and tissues were poorly correlated. ORM is synthesized in cells and secreted into the circulation. Tissue ORM expression represents the intracellular level, and serum ORM expression represents the secreted level. The poor correlation likely indicates a nonlinear release of ORM from tissues.

### Ovariectomy increased ORM level and muscle endurance

**Figure 4:**
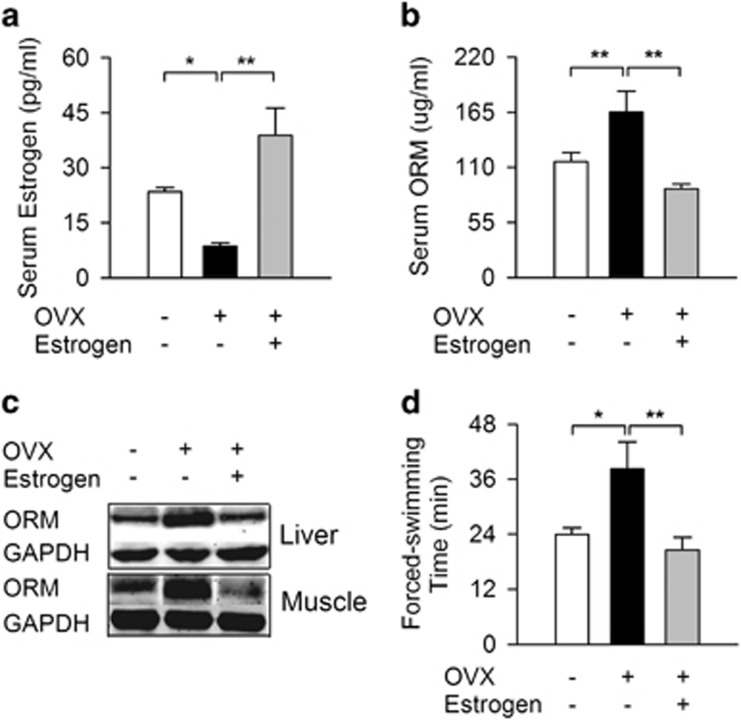
Ovariectomy (OVX) increases orosomucoid (ORM) level and muscle endurance. (**a**) Serum estrogen level in female Sprague-Dawley rats with sham operation, OVX without or with E2 replenishment (0.5 mg kg^−1^ of E2 was subcutaneously injected for 15 days after OVX surgery). (**b**, **c**) ORM level in serum (**b**), liver and muscle (**c**) of rats treated as mentioned in (**a**). (**d**) Forced-swimming times of rats treated as mentioned in (**a**). *N*=8/group. Data are presented as the means±s.e.m. *P<0.05 and **P<0.01 by one-way analysis of variance (ANOVA) with least significant difference (LSD) *t*-test.

Hormonal factors, particularly estrogen, may be responsible for the gender difference. Ovariectomy was performed on female Sprague-Dawley rats to examine the possible relationship between endogenous estrogen, ORM expression and muscle endurance. Serum estrogen concentrations decreased significantly 1 month after ovariectomy (Figure 4a), and this decrease was accompanied with elevated ORM level in sera (Figure 4b), liver and muscle (Figure 4c). Swimming time also increased significantly in ovariectomized rats (Figure 4d). Three naturally occurring estrogens in circulation are estrone, 17β-estradiol (E2) and estriol, and E2 is the most potent and most commonly used bioactive estrogen in experiments.^35^ Subcutaneous E2 injections (0.5 mg kg^−1^) for 15 days reversed the above-mentioned effects in ovariectomized rats, indicating a causal relationship between estrogen, ORM and muscle endurance.

### Exogenous estrogen suppressed ORM expression and muscle endurance

**Figure 5:**
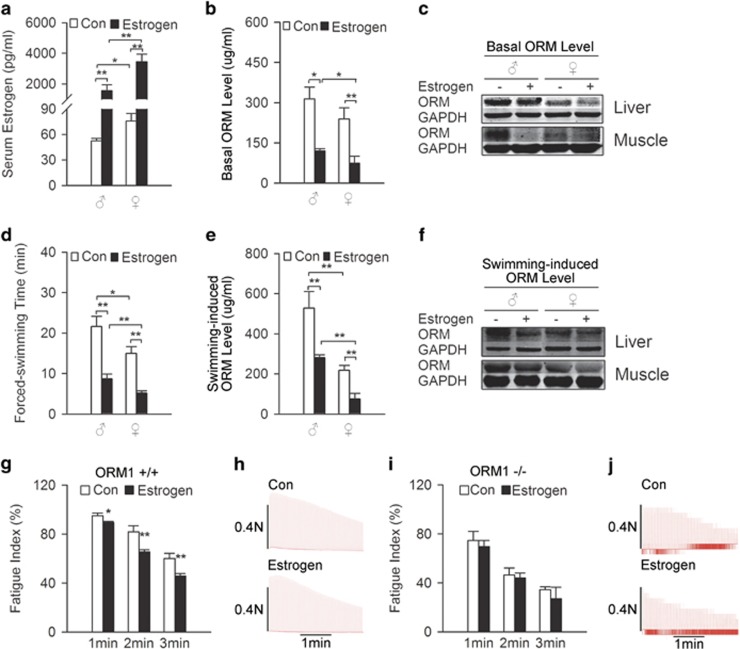
Exogenous estrogen suppresses orosomucoid (ORM) expression and muscle endurance. (**a**) Estrogen level in the serum of male and female C57BL/6J mice treated with or without subcutaneous injections of E2 for 15 days at a dose of 0.5 mg kg^−1^ (*n*=7−9/group). (**b**, **c**) Basal ORM level in serum (**b**), liver and muscle (**c**) of mice treated as mentioned in (**a**) (*n*=3−8/group). (**d**) Swimming times of mice treated as mentioned in (**a**) (*n*=10–16/group). (**e**, **f**) Swimming-induced ORM levels in serum (**e**), liver and muscle (**f**) of mice treated as mentioned in (**a**) (*n*=4−7/group). (**g**, **h**) Representative records of electrically evoked contractions of soleus muscle isolated from wild-type mice treated as mentioned in (**a**) (*n*=6−9/group). (**i**, **j**) Representative records of electrically evoked contractions of soleus muscle isolated from ORM1-deficient mice treated as mentioned in (**a**) (*n*=6/group). Data are presented as the means±s.e.m. For (**e**), **P<0.01 by two-way analysis of variance (ANOVA) with least significant difference (LSD) *t-*test. For (**a**, **b**, **d**), *P<0.05 and **P<0.01 by Friedman *M*-test.

E2 (0.5 mg kg^−1^) was administered via subcutaneous injection for 15 days (Figure 5a) in wild-type and ORM1^−/−^ knockout mice to further investigate the effect of exogenous estrogen on ORM expression and muscle endurance. Figures 5b and c show that estrogen treatment significantly decreased basal ORM levels in the serum, liver and muscle of male and female mice. These mice exhibited significantly decreased swimming times (Figure 5d) and weaker ORM induction in response to swimming-induced fatigue (Figures 5e and f). This long-term estrogen treatment also significantly decreased soleus muscle fatigue index in wild-type mice (Figures 5g and h) but not in ORM1 knockout mice (Figures 5i and j).

### Estrogen inhibited ORM expression in muscle cells and liver cells *in vitro*

**Figure 6:**
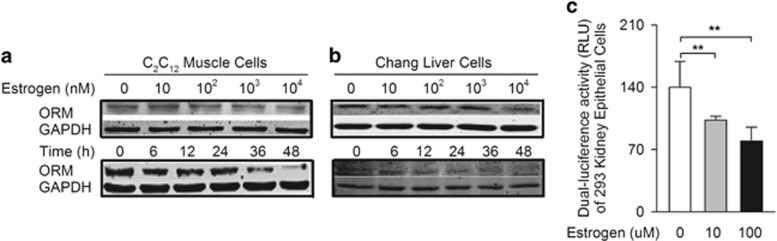
Estrogen inhibits orosomucoid (ORM) expression of muscle cells and liver cells *in vitro*. (**a**, **b**) Representative western blot of ORM in the mouse C2C12 muscle cell line (**a**) and human Chang liver cell line (**b**) treated with the indicated doses of estrogen for 48 h or 50 μM estrogen for the time indicated. (**c**) Luciferase activity in 293 kidney epithelial cells transfected with the ORM luciferase reporter plasmid and the indicated doses of estrogen for 24 h (*n*=5/group). Western blots are representative of three independent experiments. Data are presented as the means±s.e.m. **P<0.01 by one-way analysis of variance (ANOVA) with least significant difference (LSD) *t-*test.

The effect of estrogen on ORM expression was further examined *in vitro*. E2 treatment decreased ORM protein expression in a dose- and time-dependent manner in C2C12 muscle cells and Chang liver cells (Figures 6a and b). It also decreased ORM mRNA expression (Supplementary Figures S1a–d). Luciferase reporter results demonstrated that estrogen treatment attenuated mouse ORM promoter activity, indicating a negative effect of estrogen at the ORM transcriptional level (Figure 6c).

### Estrogen suppressed ORM expression via an ER-dependent pathway

**Figure 7:**
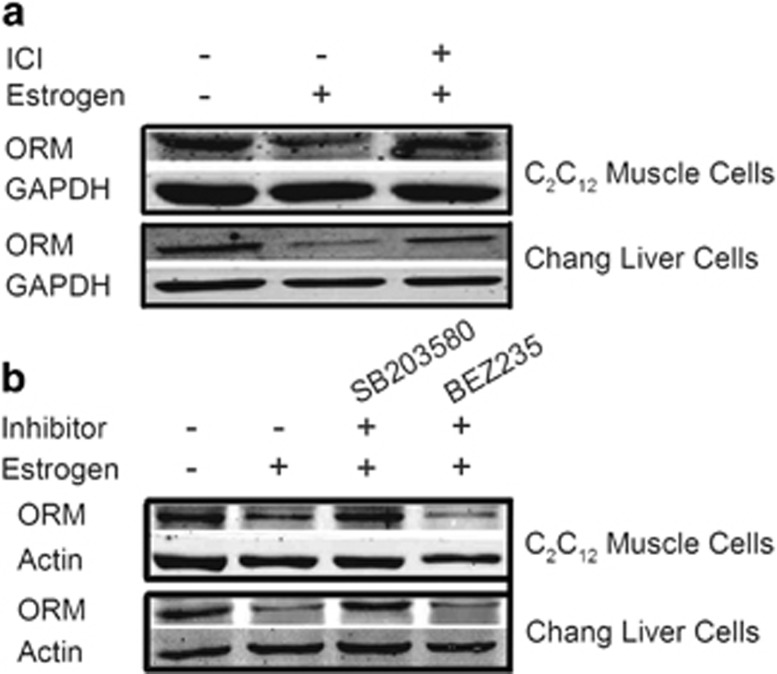
Estrogen suppresses orosomucoid (ORM) expression via the estrogen receptor (ER) and p38 mitogen-activated protein kinase (MAPK) pathway. (**a**) Representative western blot of ORM in the mouse C2C12 muscle cell line and human Chang liver cell line treated with vehicle or 50 μM estrogen for 48 h in the presence or absence of 50 μM ICI 182780 (a selective ER antagonist). (**b**) Representative western blot of ORM in the mouse C2C12 muscle cell line and human Chang liver cell line treated with vehicle or 50 μM of estrogen for 48 h in the presence or absence of 50 μM SB203580 (a p38 MAPK inhibitor) or 1 μM BEZ235 (a phosphatidylinositol 3-kinase (PI3K) inhibitor). Western blots are representative of three independent experiments.

**Figure 8:**
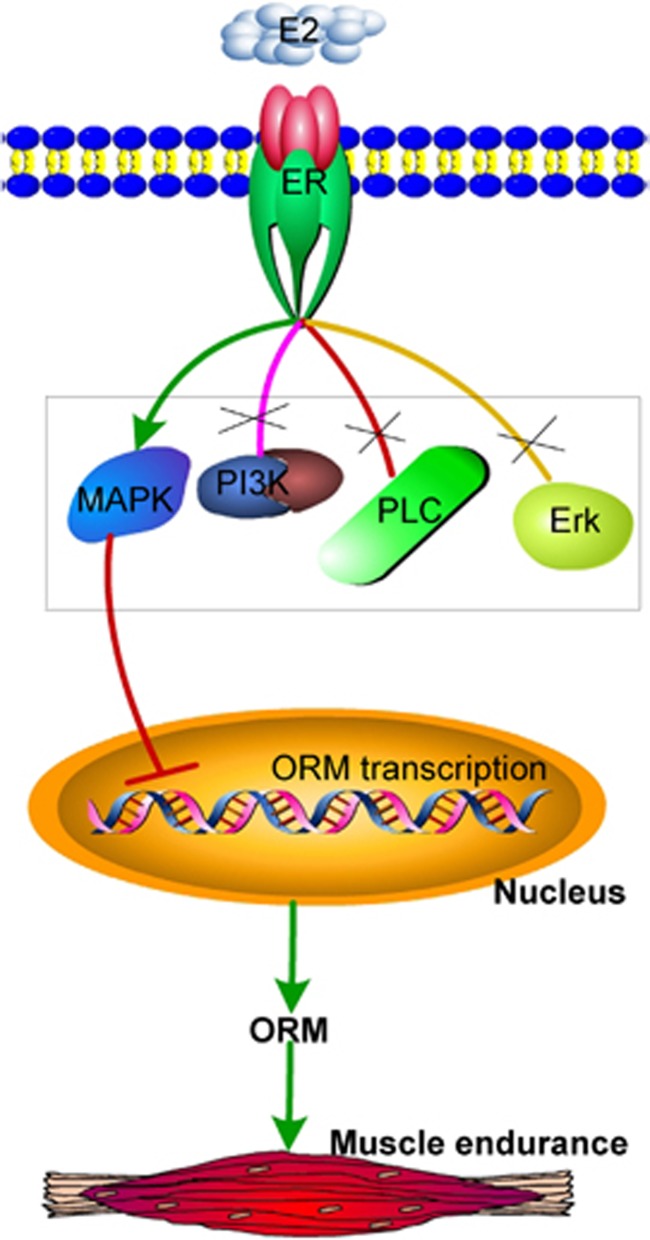
Proposed working model for estrogen regulation of orosomucoid (ORM) in skeletal muscle endurance. Estrogen negatively altered ORM transcription via an estrogen receptor-activated p38 mitogen-activated protein kinase (MAPK) pathway to influence skeletal muscle endurance.

The effect of estrogen on ORM transcription was investigated further. Most estrogen-mediated signaling pathways are ER dependent, but ER-independent pathways also exist.^36^ ER blockade using the selective ER degrader ICI 182780 completely abolished the inhibitory effect of estrogen on ORM expression in C2C12 muscle cells and Chang liver cells (Figure 7a and Supplementary Figures S2a and b). ER activation initiates its signal pathways in the nucleus or at the plasma membrane. Nuclear-initiated ER signaling directly regulates target gene transcription via binding to the ER response element, and the membrane-initiated pathway is mediated via the activation of different protein kinases, such as p38 MAPK, PI3K, extracellular signal-regulated kinase, and phospholipase C.^36, 37^ Our results demonstrated that the p38 MAPK inhibitor SB203580 blocked the inhibitory effect of E2 on ORM expression in these cells, and the dual ATP-competitive PI3K and mammalian target of rapamycin inhibitor BEZ235, the extracellular signal-regulated kinase-1/2 inhibitor SCH772984 and the phospholipase C inhibitor U73122 did not block this effect (Figure 7b and Supplementary Figures S2c–d and S3). These data suggest that estrogen regulates ORM expression via the ER and its downstream p38 MAPK pathway (Figure 8).

## Discussion

Historical studies of muscle fatigue were performed exclusively in males, and an understanding of potential gender differences in muscle fatigue has been a major focus of investigation only in the past few decades. However, the observed gender differences in muscle fatigue are controversial. Some researchers find no sex-based differences in muscle fatigue, and other studies report that females exhibit greater fatigue resistance than males.^38, 39^ More recently, studies demonstrated that men exhibit greater muscle endurance (less fatigability) than women.^5, 6, 13, 14, 15, 16^ The lack of consensus on gender fatigability is likely the result of a variety of factors, including the contraction protocols used to produce fatigue, the index of measurement (that is, endurance time vs decrease in force), age, hormones and other individual indexes of subjects.^40^ Peripheral factors (such as muscle mass, metabolism and perfusion) and central factors (such as force output to motoneurons) for the gender difference in muscle endurance were investigated, but it is not completely understood.

We first verified that females exhibited poorer performance and muscle endurance in different rodent fatigue models *in vivo* and *in vitro*. ORM is an endogenous anti-fatigue protein that is induced by fatigue and acts on muscle CCR5 to increase glycogen content and muscle endurance.^2, 24^ Therefore, we investigated its role in the gender difference of fatigue and muscle endurance. We found that the poorer performance and muscle endurance in females was accompanied by weaker ORM induction in physical fatigue (forced-swimming and treadmill-running) and mental fatigue (sleep-deprivation) than that in males, suggesting that female hormone regulates ORM.

ORM is an acute-phase protein that is upregulated by glucocorticoids, tumor necrosis factor-α, interleukin (IL)-1, IL-8, IL-11 and IL-6 cytokines in liver cells from humans, rats, mice and rabbits, and retinoic acids, phenobarbital, farnesoid X receptor, rifampicin and macrolide antibiotics.^17^ The present study found that ORM could be downregulated by estrogen at the transcriptional level via an ER-dependent pathway in liver and muscle tissues. This inhibition of ORM by estrogen may be responsible for the weaker muscle endurance in females. Whether other sex hormones (such as testosterone and progesterone) affect ORM expression is also worthy of future investigation. Notably, basal serum ORM levels were similar between males and females, but the isolated soleus muscle of females exhibited decreased muscle endurance in response to continuous electrostimulation (Figures 1e and 2) that might be closely related to the lower basal ORM level in female muscles under physiological conditions (Figure 5c).

The importance of muscle glycogen on muscle performance was demonstrated in a variety of studies, including our previous studies.^2, 41, 42^ Glycogen depletion is a major factor associated with the onset of fatigue, and most athletes seek methods to increase their muscle glycogen stores in endurance sports.^43^ We found that ORM administration significantly increased muscle glycogen content in mice.^2^ Exercise-trained women exhibit a reduced capacity to store supranormal muscle glycogen levels in response to a diet rich in carbohydrates, and 17β-estradiol administration to men significantly reduces basal muscle glycogen concentration.^44^ ORM may also mediate the effect of estrogen on muscle glycogen content in human skeletal muscle, but this effect requires further investigation.

The isometric twitch tensions of skeletal muscle are significantly higher in ovariectomized rats than in sham-operated rats or ovariectomized rats that receive estradiol,^45^ indicating a negative role of long-term estrogen in muscle fatigue. Notably, acute 17β-estradiol treatment of *in vitro* frog skeletal muscle facilitated the fatigue response to repetitive tetanus stimuli with high frequency, and this direct and quick effect may be related to an increase in the imbalance of Ca^2+^ turnover in the cytoplasm.^46^ Estrogen also influences glucose uptake via regulation of GLUT4 expression in muscles.^47^ GLUT4-deficient mice exhibit a hastened onset of muscle fatigue.^48^ These results indicate a diverse regulation of estrogen in muscle endurance beyond ORM regulation.

Notably, estrogen also regulates ORM glycosylation. Wells *et al.*^49^ reported that a decrease in the carbohydrate portion of ORM was association with an increase in female sex hormone levels. Brinkman-Van der Linden *et al.*^50^ reported that oral estrogen treatment increased the degree of branching and decreased fucosylation and sialyl Lewis X expression compared with control individuals. Changes in the carbohydrate composition of ORM may alter its function because the interaction of ORM and CCR5 is partially dependent on ORM glycosylation.^51^ Whether the estrogen-induced glycosylation changes alters the effect of ORM in muscle endurance should be further examined.
