# Supplementary material for: Estrogen weakens muscle endurance via estrogen receptor-p38 MAPK-mediated orosomucoid (ORM) suppression
Source: Exp Mol Med. 2018 Mar 30;50(3):e463–. doi: 10.1038/emm.2017.307 (PMC5898901; doi:10.1038/emm.2017.307)
Supplement: Supplementary Information [file emm2017307x1.docx]

**Estrogen weakens muscle endurance via estrogen receptor-p38 MAPK-mediated orosomucoid (ORM) suppression**

Yang Sun^1^^, 3^, Zhen Qin^1, 3^, Jing-Jing Wan^1^, Peng-Yuan Wang^1^, Yi-Li Yang^2^, Jian-Guang Yu^1^, Bo-Han Hu^1^, Ding-Feng Su^1^, Zhu-Min Luo^1^, Xia Liu^1^

^1^Department of Pharmacology, School of Pharmacy, Second Military Medical University, Shanghai 200433, P. R. China; ^2^Suzhou Institute of Systems Medicine, Center for Systems Medicine, Chinese Academy of Medical Sciences, Suzhou 215123, P. R. China

^3^Co-first author.

Correspondence: X Liu or ZM Luo, Department of Pharmacology, School of Pharmacy, Second Military Medical University, Shanghai 200433, P. R. China

Email: [lxflying@aliyun.com](mailto:lxflying@aliyun.com) or 285416709@qq.com

Tel.: +86-21-8187-1278

Fax: +86-21-6549-3951

**Running title:** Estrogen inhibits ORM and muscle endurance


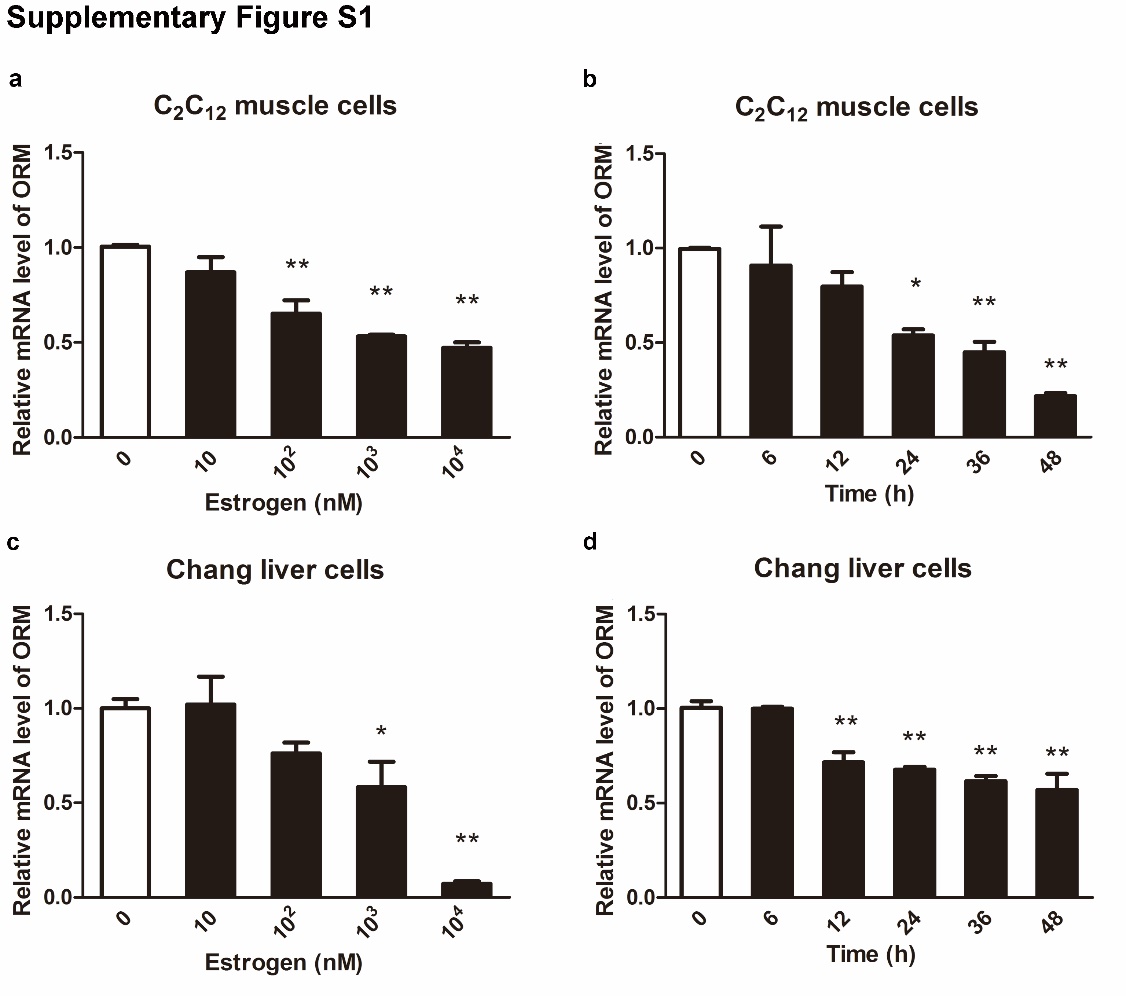


**Supplementary Figure S1** Estrogen inhibits ORM expression in vitro muscle cells and liver cells. (**a**, **b**) Relative mRNA level of ORM in mouse C2C12 muscle cell line treated with the indicated doses of estrogen for 48 h (**a**) or 50 μM of estrogen for the time indicated (**b**) (n=3/group). (**c**, **d**) Relative mRNA level of ORM in human Chang liver cell line treated with the indicated doses of estrogen for 48 h (**c**) or 50 μM of estrogen for the time indicated (**d**) (n=3/group). Data are presented as mean ± SEM. *P<0.05, **P<0.01 versus control by one-way ANOVA with LSD-t test.


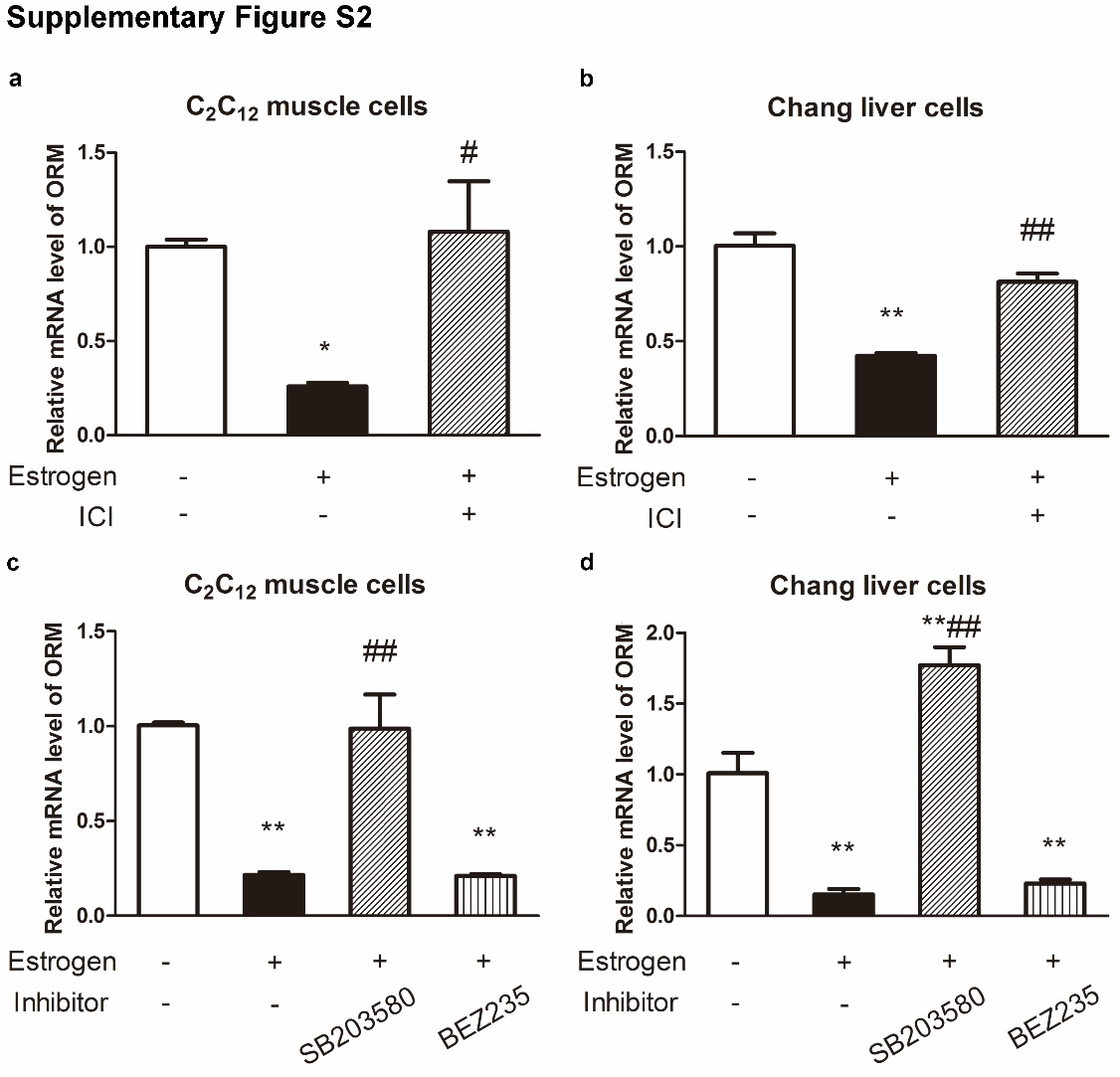


**Supplementary Figure S2** Estrogen suppresses ORM expression via estrogen receptor and p38 MAPK pathway. (**a**, **b**) Relative mRNA level of ORM in mouse C2C12 muscle cell line (**a**) or in human Chang liver cell line (**b**) treated with vehicle or 50μM estrogen for 48 h in the presence or absence of 50μM ICI 182,780 (a selective ER antagonist) (n=3/group). (**c**, **d**) Relative mRNA level of ORM in mouse C2C12 muscle cell line(**c**) or in human Chang liver cell line (**d**) treated with vehicle or 50μM of estrogen for 48 h in the presence or absence of 50μM SB203580 (a p38 MAPK inhibitor) or 1 μM BEZ235 (a PI3K inhibitor) (n=3/group). Data are presented as mean ± SEM and analyzed by one-way ANOVA with LSD-t test. *P<0.05, **P<0.01 versus negative control, ^#^P<0.05, ^##^P<0.01 versus estrogen group.


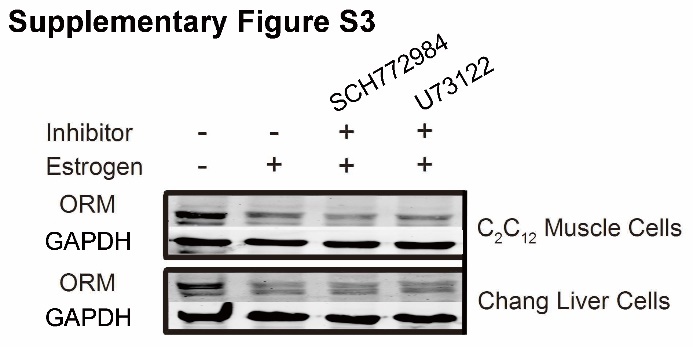


**Supplementary Figure S3** Estrogen suppresses ORM expression independent of ERK and PLC pathways. Representative western blot of ORM in mouse C2C12 muscle cell line or in human Chang liver cell line treated with vehicle or 50μM of estrogen for 48 h in the presence or absence of 0.5μM SCH772984 (a ERK1/2 inhibitor) or 10μM U73122 (a PLC inhibitor). Western blots are representative of three independent experiments.
